# The Role of Social Media in Enhancing Clinical Trial Recruitment: Scoping Review

**DOI:** 10.2196/22810

**Published:** 2020-10-26

**Authors:** Ida Darmawan, Caitlin Bakker, Tabetha A Brockman, Christi A Patten, Milton Eder

**Affiliations:** 1 Hubbard School of Journalism and Mass Communication University of Minnesota Minneapolis, MN United States; 2 Health Sciences Libraries University of Minnesota Minneapolis, MN United States; 3 Department of Psychiatry and Psychology Mayo Clinic Rochester, MN United States; 4 Center for Clinical and Translational Science Mayo Clinic Rochester, MN United States; 5 Department of Family Medicine & Community Health University of Minnesota Minneapolis, MN United States; 6 Clinical and Translational Science Institute University of Minnesota Minneapolis, MN United States

**Keywords:** social media, clinical trial, recruitment methods, enrollment methods, review

## Abstract

**Background:**

Recruiting participants into clinical trials continues to be a challenge, which can result in study delay or termination. Recent studies have used social media to enhance recruitment outcomes. An assessment of the literature on the use of social media for this purpose is required.

**Objective:**

This study aims to answer the following questions: (1) How is the use of social media, in combination with traditional approaches to enhance clinical trial recruitment and enrollment, represented in the literature? and (2) Do the data on recruitment and enrollment outcomes presented in the literature allow for comparison across studies?

**Methods:**

We conducted a comprehensive literature search across 7 platforms to identify clinical trials that combined social media and traditional methods to recruit patients. Study and participant characteristics, recruitment methods, and recruitment outcomes were evaluated and compared.

**Results:**

We identified 2371 titles and abstracts through our systematic search. Of these, we assessed 95 full papers and determined that 33 studies met the inclusion criteria. A total of 17 studies reported enrollment outcomes, of which 9 achieved or exceeded their enrollment target. The proportion of participants enrolled from social media in these studies ranged from 0% to 49%. Across all 33 studies, the proportion of participants recruited and enrolled from social media varied greatly. A total of 9 studies reported higher enrollment rates from social media than any other methods, and 4 studies reported the lowest cost per enrolled participant from social media.

**Conclusions:**

While the assessment of the use of social media to improve clinical trial participation is hindered by reporting inconsistencies, preliminary data suggest that social media can increase participation and reduce per-participant cost. The adoption of consistent standards for reporting recruitment and enrollment outcomes is required to advance our understanding and use of social media to support clinical trial success.

## Introduction

Patient recruitment continues to be a major challenge in clinical trials, with 19% of trials being terminated due to poor recruitment and another one-third needing to extend recruitment time [1,2]. Delay and termination of clinical trials has significant scientific, ethical, and financial impact on patients, researchers, and society [3]. Termination of clinical trials due to recruitment issues leads to failure to obtain the necessary evidence to assess efficacy, safety, and effectiveness of an intervention, which could subsequently delay the implementation of a more beneficial therapy or allow an existing suboptimal standard therapy to remain in practice [3,4]. From an ethical perspective, study termination exposes enrolled patients to unnecessary risks and inconvenience due to the impossibility of generating sufficient data [3]. Furthermore, delay and termination also involve human, time, and financial opportunity costs, lower researchers’ morale, and may reduce society’s trust and subsequent willingness to participate in medical research [3].

Multiple factors contribute to recruitment failure in clinical trials, including lack of funds, complex and unclear trial design, and failure to find and interest eligible participants [5]. The inability to reach potential participants may result from overestimation of prevalence, competing trials, and ineffective advertising strategies [5]. Although researchers employ multiple strategies to improve clinical trials recruitment, evidence on best practices is lacking.

In recent years, researchers have increasingly augmented traditional methods, such as newspaper, radio or television advertisements, flyers, and signs on buses, with social media strategies to help accelerate enrollment and achieve their recruitment targets. Social media may offer distinct benefits compared with traditional methods due to its ability to target specific patient segments using customized messages that may resonate better with the target segments [6]. Additionally, several social media platforms have a higher proportion of users from minority groups (eg, African American use of Twitter), which can facilitate reach and diversity of recruitment for trials [6].

Research evaluating the effectiveness of social media in enhancing clinical trial recruitment in various settings has produced conflicting results [7-10]. Some studies find social media more effective and less costly than traditional methods [7,8], while others do not [9,11]. A scoping review on the use of social media in health research recruitment published in 2016 also yielded inconclusive findings [12]. Differences in the results reported appear to be due to the ways researchers defined social media, the types of studies included in the review, and variation in how studies compared and assessed recruitment and enrollment through social media and traditional methods.

In this study, we define social media as “a group of Internet-based applications that build on the ideological and technological foundations of Web 2.0, and that allow the creation and exchange of User Generated Content” [13]. According to the Organization for Economic Co-operation and Development definition, user-generated content needs to be published on a website that is publicly accessible, show some creative effort, and not be created in the context of professional work [14]. This definition excludes online media supported by internet-based platforms, such as email, instant messaging, regular websites, replications of existing content, and work created by companies for commercial use. We define traditional methods as offline (non–internet-based) platforms, such as television, print, and in-person recruitment. By using generally accepted definitions of social and traditional media, we address the heterogeneity observed in a prior scoping review and more clearly articulate the basis for including studies in this scoping review.

The prior scoping review included both observational and interventional studies [12], which potentially confounded the results. To avoid potential incommensurability across studies, this review includes only research studies that meet the National Institutes of Health’s definition of a clinical trial [15]. Beyond refinements to the inclusion criteria, new studies comparing the use of social media and traditional methods for clinical trial recruitment have been published. As a result, two-thirds of the studies included in this scoping review were not evaluated in the prior scoping review.

Our scoping review examined the literature on the use of social media in conjunction with traditional methods in clinical trials to improve recruitment outcomes (success rate and cost). Specifically, this review addressed the following questions: (1) How is the use of social media, in combination with traditional approaches to enhance clinical trial recruitment and enrollment, represented in the literature? (2) Do the data on recruitment and enrollment outcomes presented in the literature allow for comparison across studies?

## Methods

### Review Method and Search Strategy

A scoping review was performed and reported using the Preferred Reporting Items for Systematic Reviews and Meta-Analyses extension for scoping reviews (PRISMA-ScR) guidelines, and the checklist is provided in Multimedia Appendix 1 [16]. The protocol can be accessed through the Open Science Framework website [17]. Since this study focused on previously published literature and thus did not involve direct contact with human participants, institutional review board approval was not required [18].

We performed a comprehensive search in July 2019 using the following databases: PubMed; MEDLINE, EMBASE, and PsycInfo via Ovid; Cochrane Library via Wiley; Scopus; and Web of Science Core Collection. A combination of natural language and controlled vocabulary was employed in accordance with Methodological Expectations of Cochrane Intervention Reviews guidelines [19]. A complete search strategy is available in Multimedia Appendix 2. To ensure that we did not overlook potentially relevant items, we also reviewed reference lists of related systematic and scoping reviews and included studies. Results were compiled and deduplicated in EndNote (version X.9; Clarivate Analytics).

### Inclusion and Exclusion Criteria

Papers employing strategies according to the stated definitions of social media and traditional methods were included. Papers also needed to be a clinical trial and describe at least one of the following outcomes: number of participants recruited or enrolled, cost of recruitment, or length of recruitment. Papers that contained no original data (editorials, letters to editors, opinions, conference proceedings, comments, systematic reviews), that recruited health care professionals as participants, or that did not report outcomes of interest were excluded.

### Screening

Papers were screened in 2 stages: (1) title and abstract screening and (2) full-text screening using the aforementioned inclusion and exclusion criteria. Two independent screeners reviewed papers at both the title and abstract stage and the full-text stage (ID, CB, and TAB). We resolved discrepancies through discussion and consensus or through the intervention of a third party when necessary. Study screening was facilitated using Rayyan (Qatar Computing Research Institute).

### Data Charting Process

Two independent reviewers charted data using standardized collection forms created in REDCap (Vanderbilt University). Again, discrepancies were resolved by discussion to achieve consensus. Data charted included study characteristics (goal of intervention, disease, mode of intervention), recruitment methods (social media platforms, traditional methods, other online methods), participant characteristics (age, sex, ethnicity, geographic location, type of residential area), and recruitment outcomes (number of participants recruited via each method, cost of recruitment, length of recruitment). We encountered differences in the way researchers described recruitment versus enrollment outcomes across studies. As a result, we defined recruitment as the first contact between prospective participants and study staff and enrollment as when participants were enrolled in the study after they signed informed consent. We extracted both recruitment and enrollment data when available.

## Results

### Paper Selection

**Figure 1:**
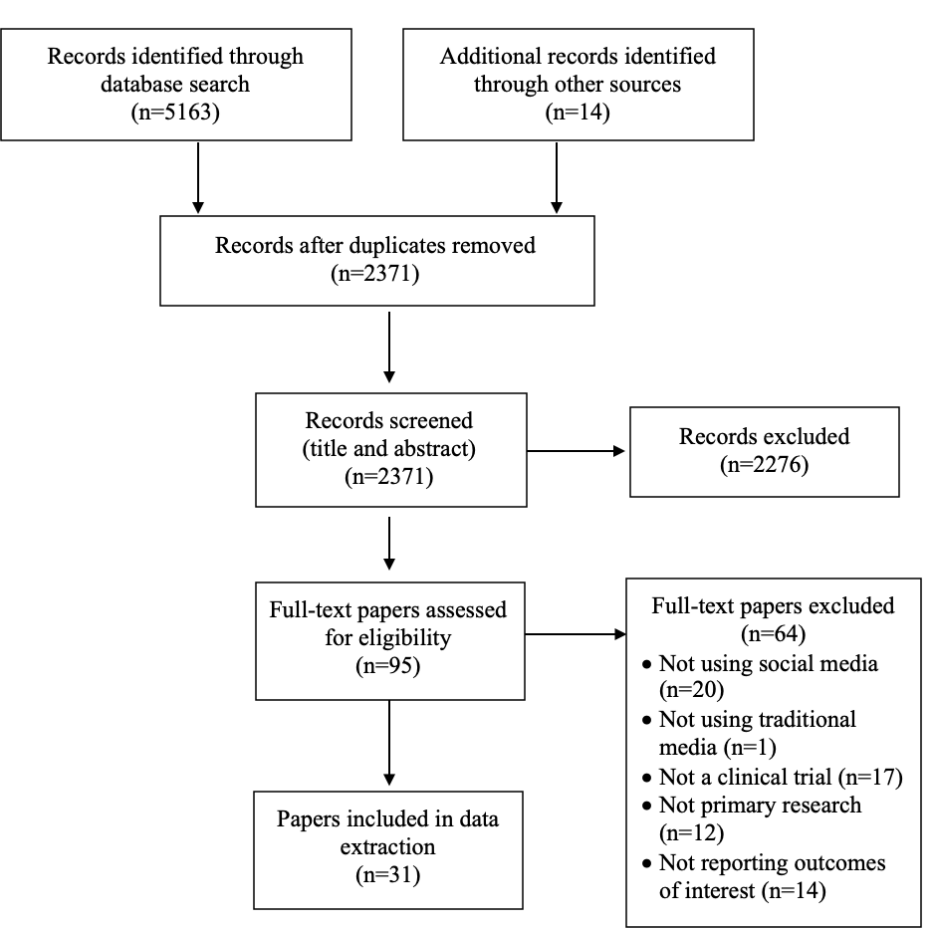
PRISMA flow diagram.

Our search strategy identified a total of 5177 papers. After removing duplicates (n=2806), the titles and abstracts of 2371 papers were screened against the exclusion criteria, resulting in 95 papers for full-text screening. From this, 64 papers did not meet inclusion criteria (Figure 1); the remaining 31 papers were included in this review and described in detail below.

### Study Characteristics

Of the 31 papers, 29 reported on a single study and 2 included reports on 2 studies each. This review identified a total of 33 studies that reported using social media and traditional methods in recruitment to clinical trials. The 33 studies described interventions for treatment (n=19) and prevention (n=14). A total of 19 studies consisted of in-person visits only, 10 were online only, and 4 consisted of in-person and online participation. Study diseases or conditions focused on lifestyle and behavior (eg, weight loss, smoking), neurology, obstetrics/gynecology, and HIV. Most studies (32/33) recruited adults (18 years or older), with 5 studies focusing on older adults and seniors; 1 study recruited adolescents. Detailed characteristics of included studies are presented on Table 1.

**Table 1:** Characteristics of included studies.

Study authors (year)	Purpose of intervention	Mode of intervention	Diseases or conditions	Participant’s age (years)
Abbate et al (2017) [20]	Treatment	Online	Smoking	18+
Adam et al (2016) [21]	Prevention	In-person	Weight gain and diet	23-40
Alley et al (2016) [22]	Prevention	Online	Physical activity	18+
Bracken et al (2019) [23]	Prevention	In-person	Type 2 diabetes	50-74
Buckingham et al (2017) [24]	Prevention	In-person	HIV	18+
Burrell et al (2012) [25]	Prevention	In-person	HIV	18+
Cowie et al (2018) [26]	Treatment	In-person	Alzheimer disease	60+
Frandsen et al (2014) [27]	Treatment	In-person	Smoking	18+
Frandsen et al (2016) [9]	Treatment	In-person	Smoking	18+
Guthrie et al (2019) [28]	Treatment	In-person	Postmenopausal vulvovaginal symptoms	45-70
Heffner et al (2013) [29]	Treatment	Online	Smoking	18+
Huesch et al (2018) [30]	Treatment	In-person	Bipolar disorder	18+
Ince et al (2014) [31]	Treatment	Online	Depressive symptoms	18+
Jones et al (2012) [32]	Prevention	In-person	Physical activity	16-18^a^
Jones et al (2017) [33]	Prevention	Online	HIV	18-29
Juraschek et al (2018) [34]	Prevention	Both	Cancer survivors	18+
Kayrouz et al (2016) [10]	Treatment	Online	Anxiety, depression	18-70
Kendall et al (2018) [35]	Treatment	In-person	Chronic dizziness, chronic neck pain	65-85
Kira et al (2016) [36]	Prevention	In-person	Smoking	16+
Kuhn et al (2017) [37]	Treatment	Online	PTSD^b^	18+
Nash et al (2017) [38]	Treatment	In-person	Hypertension	18-69
Partridge et al (2015) [39]	Prevention	Both	Weight gain	18-35
Rabin et al (2013) [40]	Prevention	Online	Physical activity	18-39
Raviotta et al (2016) [41]	Prevention	Both	HPV^c^	18-25
Rounds et al (2019) [42]	Treatment	Both	Obesity	18-65
Sanchez et al (2018a) [43]	Treatment	In-person	ARHL^d^	18+
Sanchez et al (2018b) [43]	Treatment	In-person	ARHL	50-89
Shere et al (2014) [44]	Treatment	In-person	Pregnancy	18-45
Usadi et al (2015a) [45]	Treatment	In-person	Infertility	18-40
Usadi et al (2015b) [45]	Treatment	In-person	Infertility	18-40
Volkova et al (2017) [46]	Prevention	Online	Healthy food purchase	18+
Waltman et al (2019) [47]	Prevention	In-person	Bone loss	19+
Watson et al (2018) [48]	Treatment	Online	Smoking	18+

^a^Participants were 11th-grade students. Age range is estimated.

^b^PTSD: posttraumatic stress disorder.

^c^HPV: human papillomavirus.

^d^ARHL: age-related hearing loss.

### Recruitment Methods

Facebook was the most commonly used social media platform (31/33), followed by Twitter (6/33). The majority of studies (21/33) used a single social media platform and the remainder used two or more social media platforms, with one study reporting the use of 4 platforms. Facebook use occurred across all studies reporting the use of more than one social media platform.

Studies that reported using Facebook for their recruitment used a variety of Facebook features: Facebook ads, Facebook pages (including boosted posts on a page) [10,36], or sending a friend invite to prospective participants using a Facebook account [32]. One study used untargeted Facebook ads, then switched to targeted Facebook ads that showed ads only to participants who met the study criteria [22]. Several studies started using social media after other recruitment methods were deployed [28,31,38,44], while others used social media at the beginning of their recruitment period.

The most commonly used traditional method was print (32/33), followed by in-person venues (17/33) and referrals from health care professionals (16/33). A total of 23 studies used other online media, such as website ads (15/23), emails (11/23), and Craigslist (8/23).

### Recruitment and Enrollment Rates

A total of 17 out of 33 studies reported overall enrollment rates. Of these, 9 studies achieved or exceeded their enrollment target. Of these 9 studies, 8 reported the proportion of participants enrolled through social media, which ranged from 0% to 49%. One study that achieved its enrollment target reported that social media outperformed other recruitment methods [48].

About half of the studies (17/33) reported both recruitment and enrollment rates from social media, 3 studies reported recruitment rates only, 11 studies reported enrollment rates, and 2 did not report either (Table 2). The proportion of participants recruited and enrolled from social media varied greatly from study to study (Table 2) and across study types (Table 3). Studies with a high proportion (50% or greater) of participants enrolled from social media recruited adult participants. Studies recruiting seniors [26,35,43] and the 1 study involving adolescents [32] enrolled the majority of participants from traditional methods (Table 3).

**Table 2:** Reported recruitment and enrollment from social media.

Study authors (year)	Participants recruited from social media, n/N (%)^a^	Participants enrolled from social media, n/N (%)^a^
Abbate et al (2017) [20]	—^b^	24/151 (16)
Adam et al (2016) [21]	45/126 (36)	25/70 (36)
Alley et al (2016) [22]	205/278 (74)	74/140 (53)^c^
Bracken et al (2019) [23]	369/19,022 (2)	16/1007 (2)
Buckingham et al (2017) [24]	598/1945 (31)	48/96 (50)
Burrell et al (2012) [25]	—	24/105 (23)
Cowie et al (2018) [26]	621/857 (72)	—
Frandsen et al (2014) [27]	—	138/266 (52)
Frandsen et al (2016) [9]	228/414 (55)	92/175 (53)
Guthrie et al (2019) [28]	461/2627 (18)	25/302 (8)
Heffner et al (2013) [29]	—	11/222 (5)
Huesch et al (2018) [30]	117/147 (80)	11/17 (65)
Ince et al (2014) [31]	227/287 (79)	75/96 (78)
Jones et al (2012) [32]	—	43/589 (7)
Jones et al (2017) [33]	940/1435 (66)	153/247 (62)
Juraschek et al (2018) [34]	24/121 (6)	4/121 (3)
Kayrouz et al (2016) [10]	—	70/81 (86)
Kendall et al (2018) [35]	38/162 (23)	8/24 (33)
Kira et al (2016) [36]	1/74 (1)	1/24 (4)
Kuhn et al (2017) [37]	—	22/120 (18)
Nash et al (2017) [38]	—	—
Partridge et al (2015) [39]	20/1181 (2)	5/250 (2)
Rabin et al (2013) [40]	11/73 (15)^d^	0/12 (0)
Raviotta et al (2016) [41]	—	44/220 (20)
Rounds et al (2019) [42]	—	3/102 (3)
Sanchez et al (2018a) [43]	4/425 (1)	0/91 (0)
Sanchez et al (2018b) [43]	N/A^e^	0/79 (0)
Shere et al (2014) [44]	—	—
Usadi et al (2015a) [45]	7/3358 (0.2)	N/A
Usadi et al (2015b) [45]	3/3727 (0.1)	N/A
Volkova et al (2017) [46]	966/2448 (40)	584/1357 (43)
Waltman et al (2019) [47]	838/3033 (28)	44/276 (16)
Watson et al (2018) [48]	—	1299/2637 (49)

^a^Some studies allowed participants to be counted in multiple recruitment/enrollment methods.

^b^Not available (not reported by original study).

^c^Includes participants enrolled from targeted Facebook ads only.

^d^Includes participants recruited from emails and Craigslist.

^e^N/A: not applicable. Reported as N/A due to inconsistencies in recruitment and enrollment data.

**Table 3:** Social media recruitment and enrollment rates by study type.

Study type		Studies reporting recruitment rate, n	Range of participants recruited from social media, %	Studies reporting enrollment rate, n	Range of participants enrolled from social media, %
**Intervention purpose**					
	Treatment	10	0-80	14	0-86
	Prevention	10	1-74	14	0-62
**Disease or condition type**					
	Lifestyle-related	6	1-74	13	0-53
	Neurological	4	23-80	5	18-86
	OB/GYN^a^	4	0-28	2	8-16
	HIV	2	31-66	3	23-62
	Others	4	1-6	5	0-20
**Mode of intervention**					
	In-person	14	0-80	14	0-65
	Online	4	15-79	10	0-86
	Both	2	2-6	4	2-20
**Participant age group**					
	Adults (18+ years)	15	0-80	23	0-86
	Older adults/seniors	5	1-72	4	0-33
	Adolescents	0	—^b^	1	7

^a^OB/GYN: obstetrics/gynecology.

^b^Not available (not reported by original study).

Out of 20 studies that reported recruitment rates by method, 7 reported higher recruitment rates from social media than from any other methods. Similarly, out of 28 studies that reported enrollment rates by method, only 9 reported higher enrollment rates using social media than any other methods (Table 4). Among the 9 studies that reported the highest enrollment rates from social media, 6 studies were for treatment (3 lifestyle-related conditions, 3 neurological) and 3 were for prevention (2 HIV, 1 lifestyle). Of the 9 studies reporting enrollment, 5 involved an online intervention and the remainder used an in-person intervention. A total of 5 of these 9 studies used Facebook only, and the rest used more than one social media platform.

**Table 4:** Enrollment rates by recruitment method.^a^

Study authors (year)	Enrolled from social media, %	Enrolled from traditional methods, %	Enrolled from other online media, %
Abbate et al (2017) [20]	16	40	57
Adam et al (2016) [21]	36	64	N/A^b^
*Alley et al*^c^ (2016) [22]	53^d^	47^e^	N/A
Bracken et al (2019) [23]	2	94	1
*Buckingham et al* (2017) [24]	50	26	24
Burrell et al (2012) [25]	23	77^e^	N/A
*Frandsen et al* (2014) [27]	52	47	N/A
*Frandsen et al* (2016) [9]	53	47	N/A
Guthrie et al (2019) [28]	8	92	N/A
Heffner et al (2013) [29]	5	25	70
*Huesch et al* (2018) [30]	65	35	N/A
*Ince et al* (2014) [31]	78	2	0
Jones et al (2012) [32]	7	94	N/A
*Jones et al* (2017) [33]	62	38	N/A
Juraschek et al (2018) [34]	3	77	N/A
*Kayrouz et al* (2016) [10]	86	14	N/A
Kendall et al (2018) [35]	33	67	N/A
Kira et al (2016) [36]	4	88	N/A
Kuhn et al (2017) [37]	18	22	60
Partridge et al (2015) [39]	2	71	25
Rabin et al (2013) [40]	0	100	N/A
Raviotta et al (2016) [41]	20	80	N/A
Rounds et al (2019) [42]	3	81	16
Sanchez et al (2018a) [43]	0	19	5
Sanchez et al (2018b) [43]	0	53	4
Volkova et al (2017) [46]	43	46	11
Waltman et al (2019) [47]	16	44	1
*Watson et al* (2018) [48]	49	11	39

^a^Some studies allowed participants to be counted in multiple recruitment and enrollment methods.

^b^N/A: not applicable.

^c^Italics signify studies that reported the highest enrollment rates from social media.

^d^Includes participants enrolled from targeted Facebook ads only.

^e^Includes participants enrolled from both traditional and other online media.

### Recruitment and Enrollment Costs

A total of 20 studies reported itemized recruitment and enrollment costs. Of these, almost all studies reported the costs to place the recruitment ads in various media (19/20), and 6 reported the costs to develop these ads. Only 5 studies reported the costs of staff involved in participant recruitment and enrollment.

Of the 19 studies that reported cost per enrolled participant, only 4 [10,21,30,33] reported lower cost per enrolled participant using social media than any other methods. Recruitment methods used in these 4 studies included Facebook, Instagram, print, television, radio, in-person recruitment, and website ads. Detailed costs per enrolled participant by media type can be found in Table 5.

**Table 5:** Costs per enrolled participant by media type.^a^

Study authors (year)	Cost per enrolled participant		
	Social media	Traditional methods	Other online media
Abbate et al (2017) [20]	N/A^b^	US $8.28^c^	N/A
Adam et al (2016) [21]	Can $20.28	Can $24.15	N/A
Bracken et al (2019) [23]	N/A	Aus $594	N/A
Frandsen et al (2014) [27]	Aus $56.34	Aus $52.33	N/A
Frandsen et al (2016) [9]	Aus $42.34	Aus $21.52	N/A
Guthrie et al (2019) [28]	US $593	US $356	N/A
Heffner et al (2013) [29]	US $172.76	US $5.27-$46.98	US $26.19-$50.26
Huesch et al (2018) [30]	US $18	US $635	N/A
Ince et al (2014) [31]	€5.33	N/A	N/A
Jones et al (2017) [33]	US $66.46	US $149.62	N/A
Juraschek et al (2018) [34]	US $1426	US $436-$917	N/A
Kayrouz et al (2016) [10]	US $37	US $40	N/A
Kendall et al (2018) [35]	N/A	Aus $2141^c^	N/A
Nash et al (2017) [38]	Aus $45.15-$176	N/A	N/A
Partridge et al (2015) [39]	Aus $945.33	Aus $144.52-$212.51	Aus $11.98-$571.45
Raviotta et al (2016) [41]	US $110	US $61	N/A
Volkova et al (2017) [46]	NZ $5^d^	NZ $4-$179	NZ $4
Waltman et al (2019) [47]	US $119.38	US $29.36-$926.90	US $1000
Watson et al (2018) [48]	US $40.51	US $20.30	US $13.95-34.71

^a^Currency exchange rates of Can $1=US $0.75, Aus $1=US $0.72, €1=US $1.17, and NZ $1=US $0.66 were applicable at the time of publication.

^b^N/A: not applicable.

^c^Cost per enrolled participant across all media.

^d^Includes cost using Craigslist.

## Discussion

### Principal Findings

This scoping review examines literature describing the use of social media and traditional methods as well as other online media in clinical trial recruitment. We found that using social media resulted in the highest recruitment rates in 7 of 20 studies and the highest enrollment rate in 9 of 28 studies. We also found that social media resulted in the lowest cost per enrolled participant in 4 of 19 studies. However, the data reported about social media outcomes varied greatly across studies, obscuring our ability to evaluate if particular studies might benefit more from using social media.

Our review discovered a lack of consistency in defining and reporting recruitment and enrollment data across studies. Several studies seemed to use these terms interchangeably, and some failed to specify which data they reported (eg, when consent occurred). Furthermore, we noted inconsistencies resulting from the varied use of social media, even within a platform, and the opportunistic use of social media by several studies that started using social media only after traditional methods failed to achieve sufficient interest and enrollment. We also observed that some studies did not indicate how many participants were counted as being reached by more than one recruitment or enrollment method. Lastly, we observed inconsistencies in cost reporting, with some studies reporting itemized costs and others reporting aggregated costs. Collectively, these inconsistencies contributed to the variations in describing the outcomes of using social media and traditional methods to support clinical trial recruitment and enrollment. The inconsistencies in reporting also rendered comparison of data across the studies included in this scoping review impossible.

To address these inconsistencies, we recommend that future studies use the terms “recruitment” and “enrollment” in alignment with the definitions used by the Food and Drug Administration’s advisory committee [49]. The term “recruitment” should report on all interactions with potential study participants prior to obtaining informed consent, while the term “enrollment” should report only on the total number of individuals who provide informed consent. Studies should report recruitment using as the denominator the total number of participants reached and as numerators the number of study participants reached through each method. Enrollment rates should be calculated using as the denominator the total number of participants providing informed consent and as numerators the number of participants enrolled through each recruitment method. Additionally, screening that results in the exclusion of participants after they provide informed consent should be reported separately, enabling a more accurate count and cost of the individuals actively participating in the intervention. Using consistent definitions of key terms across research studies will allow researchers to improve the clarity and comparability of clinical trial management data. Establishing reporting standards will also allow researchers to adopt evidence-based recruitment strategies by choosing optimal recruitment methods to reduce cost and timelines [50].

To facilitate shared learning based on the experience of prior research, we further recommend that future studies report in detail their approaches to using each social media platform (eg, Facebook ad, Facebook page) and specify the number of participants recruited or enrolled from multiple methods, which will allow for more accurate response analyses and cost estimates. As the ability to capitalize on existing social media relationships may influence both the success of recruitment and enrollment as well as their subsequent costs, researchers should consider explicitly stating whether their social media recruitment strategies are related to a larger social media initiative.

When adding social media to existing recruitment methods, researchers should report both the incremental costs of social media and the personnel cost to develop and monitor posted content. Furthermore, researchers are encouraged to report trade-offs between cost and time when analyzing the effectiveness of each recruitment method. For example, social media may result in higher cost per enrolled participant but reduce the time to enroll participants. Although we recognize the complexity in analyzing and comparing cost in different time increments and the potential for historical trends to confound results, such reporting may provide deeper insights on different recruitment methods. Such analyses may help researchers, organizations, and corporations determine whether to build social media expertise directly into their research teams to optimize the full extent of social media capabilities or to engage social media experts as consultants. The costs associated with such arrangements should be identified and, ideally, reported.

### Comparison With Prior Work

The mixed results on the use of social media found in this study are similar to those found in a prior scoping review [12]. While the prior scoping review attributed the different success rates to the effort researchers put into the use of social media as a recruitment method, we found that the variations in the use of social media and the inconsistencies in the way outcomes were reported prevented comparisons across studies. Having explored the ways researchers used and reported the use of social media in clinical trial recruitment, this study advances recommendations for achieving data consistency in reporting, which would facilitate comparison across results.

Similar to the findings from a systematic mapping study conducted by Frampton et al [51], we found that the use of social media to recruit participants involved a variety of diseases and health conditions. We also failed to find many studies involving minority populations (eg, African American or lesbian, gay, bisexual, transgender, and queer populations). As a result, we are unable to provide any insights on the use of social media to recruit diverse populations when compared with traditional recruitment methods.

### Limitations

Our scoping review is limited in its examination of data from clinical trials that combined social media and traditional methods in their recruitment strategies because we cannot guarantee that the review identified all relevant studies. We also cannot claim this review is comprehensive, as research not organized or reported as a clinical trial may inform reasoning about incorporating social media into the research recruitment armamentarium. The relatively recent use of social media also renders highly speculative any conclusions drawn about its appropriateness in recruitment and enrollment methods for particular diseases and conditions. We also recognize a limitation in our ability to assess the quality of the recruitment strategy, the recruitment materials, or the enrollment process. However, we advance recommendations to improve consistency in reporting on recruitment and enrollment that we believe to be necessary precursors for comparative analysis.

### Conclusions

The use of social media for clinical trial recruitment holds great promise. However, this scoping review identified continued inconsistency in reports on the use of social media for clinical trial recruitment and enrollment. We recommend that future studies incorporate the recommendations for collecting and reporting recruitment and enrollment data advanced here to facilitate comparison of study data and shared learning.

## Appendix

Multimedia Appendix 1 PRISMA-ScR checklist.

Multimedia Appendix 2 Complete search strategy.

## Abbreviations

PRISMA-ScR: Preferred Reporting Items for Systematic Reviews and Meta-Analyses extension for scoping reviews
